# The strength of co-authorship in gene name disambiguation

**DOI:** 10.1186/1471-2105-9-69

**Published:** 2008-01-29

**Authors:** Richárd Farkas

**Affiliations:** 1 Hungarian Academy of Science, Research Group on Artificial Intelligence, Aradi vertanuk tere, Szeged, Hungary

## Abstract

**Background:**

A biomedical entity mention in articles and other free texts is often ambiguous. For example, 13% of the gene names (aliases) might refer to more than one gene. The task of Gene Symbol Disambiguation (GSD) – a special case of Word Sense Disambiguation (WSD) – is to assign a unique gene identifier for all identified gene name aliases in biology-related articles. Supervised and unsupervised machine learning WSD techniques have been applied in the biomedical field with promising results. We examine here the utilisation potential of the fact – one of the special features of biological articles – that the authors of the documents are known through graph-based semi-supervised methods for the GSD task.

**Results:**

Our key hypothesis is that a biologist refers to each particular gene by a fixed gene alias and this holds for the co-authors as well. To make use of the co-authorship information we decided to build the inverse co-author graph on MedLine abstracts. The nodes of the inverse co-author graph are articles and there is an edge between two nodes if and only if the two articles have a mutual author. We introduce here two methods using distances (based on the graph) of abstracts for the GSD task. We found that a disambiguation decision can be made in 85% of cases with an extremely high (99.5%) precision rate just by using information obtained from the inverse co-author graph. We incorporated the co-authorship information into two GSD systems in order to attain full coverage and in experiments our procedure achieved precision of 94.3%, 98.85%, 96.05% and 99.63% on the human, mouse, fly and yeast GSD evaluation sets, respectively.

**Conclusion:**

Based on the promising results obtained so far we suggest that the co-authorship information and the circumstances of the articles' release (like the title of the journal, the year of publication) can be a crucial building block of any sophisticated similarity measure among biological articles and hence the methods introduced here should be useful for other biomedical natural language processing tasks (like organism or target disease detection) as well.

## Background

Biological articles provide a huge amount of information about genes, proteins, their behaviour under different conditions, and their interactions. The handling of huge amounts of unstructured data (free text) has increased in interest along with the application of automatic Natural Language Processing (NLP) techniques to biomedical articles. Named Entity (NE) recognition is the first and crucial step of an Information Extraction (IE) system and a major building block of an Information Retrieval (IR) system as well.

The task of biological entity recognition is to identify and classify gene, protein, chemical names in biological articles [1]. Taken one step further, the goal of Gene Name Normalisation (GN) [2] is to assign a unique identifier to each gene name found in a text. The GN task is challenging for two main reasons. First, although synonym (alias) lists which map gene name variants to gene identifiers exist like that given in [3], they are incomplete and they do not contain all the spelling variants [4]. On the other hand one name can refer to different entities (for example *IL-21 *can refer to the genes with EntrezGeneID 27189, 50616 or 59067). Chen et al. [5] investigated gene name ambiguity in a comprehensive empirical study and reported an average of 5% overlap on intra-species synonyms, and ambiguity rates of 13.4%, and 1.1% on inter-species and against English words respectively. In general, the Word Sense Disambiguation (WSD) approaches (for a comprehensive study, see [6]) are concerned with this crucial problem. Their goal is to select the correct sense – from a well-defined sense inventory – of a term according to its context. A special case of WSD task is the Gene Symbol Disambiguation (GSD) [7] task where the terms are gene names, the senses are genes referred by unique identifiers and the contexts are biological articles.

There are several earlier studies on general biomedical disambiguation tasks like [8-10], to name but a few. Weeber et al. [8] annotated manually a UMLS-WSD corpus for supervised learning purposes. Savova et al. [9] introduced the utility of unlabeled data in general biomedical entity disambiguation. Their unsupervised approach looked for clusters among MedLine abstracts containing the target word, based on single word and bigram, first- and second order co-occurrence information. Liu et al [10] built a train set automatically for each target term based on the co-occurrences of unambiguous synonyms in other documents. He also mentioned that disambiguation on this domain has several features which distinguish it from the general English WSD task, mainly the granularity and nature of sense distinctions. In this paper we will examine the potential utilisation of another particular fact, namely that the authors of the documents are known.

When handling the GSD task, the AZuRE system [11] automatically assigns gene names to their LocusLink IDs based on the Naive Bayes model and contextual similarity. It extracted the training sets automatically from MedLine references in the LocusLink and SwissProt databases. Schijvenaars et al [12] also generates the training set automatically from several existing databases. They build up their vector space from MeSH terms and gene names identified by string-matching then a cosine similarity metric based disambiguation is applied. The ProMiner system [13] GN system contains a disambiguation module as well. It utilises the synonyms of the target gene name which are present in the document of the test gene. In this study we present experimental results on the GSD datasets built by Xu et al [14,15]. In [14] Xu and his colleagues took the words of the abstracts, the MeSH codes provided along with the MedLine articles, the words of the texts and some computer tagged information (UMLS CUIs and biomedical entities) as features while in [15] they experimented with the use of combinations of these features. They used them to get manually disambiguated instances (training data) and applied a vector space model with cosine similarity measure between the abstracts in question and the gene profiles which were in fact the centroids of the training instances. As they pointed out, there was not any significant information gain using the texts themselves along with the manually added MeSH codes, so we decided to just use these codes along with some novel features like author information and the year of publication.

The GSD datasets for yeast, fly and mouse are generated using MedLine abstracts and the Entrez 'gene2pubmed' file [3], which is manually disambiguated [14]. The dataset for human genes was derived [15] from the training and evaluation sets of the BioCreative II GN task [16].

Our main idea here is that an author uses gene names consistently, that is they employ a gene name to refer exclusively to one gene in their publications, hence the co-authorship between articles may contain very useful information. In this study we built an inverse co-author graph on MedLine abstracts and have introduced two methods based on the graph for the GSD task. Our methods utilise unlabelled instances (which are not manually tagged on gene meanings) by looking for paths in the graph, thus it can be regarded as a semi-supervised approach in the middle of supervised (e.g. vector space based similarity models) and fully unsupervised techniques.

## Results

### The inverse co-author graph

Generalising the hypothesis that an author habitually uses a gene name to refer exclusively to one gene, we can assume that the same holds true for the co-authors of the biologist in question. But what is the situation for the co-authors of the co-authors? To answer this question – and utilise the information obtained from co-authorship in the GSD problem – we decided to use the so-called co-author graph [17]. The co-author graph represents the relationship between authors. The nodes of the graph are authors, while the edges represent mutual publications. In the GSD task we basically look for an appropriate distance (or similarity) metric between pairs of abstracts, hence we define the inverse co-author graph as a graph whose nodes are abstracts from MedLine (we usually just used their PMID and not their actual text) and there is an undirected edge between two nodes if and only if the intersection of their author sets is not empty.

### Evaluation issues

We carried out experiments utilising the inverse co-author graph on the human, fly, yeast and mouse GSD tasks. For each test instance a geneId set (the sense inventory) along with several manually disambiguated abstracts for these geneIds (the train set) were present. For details of the evaluation data sets and experimental design, see the Methods section.

We used two evaluation metrics in our study, namely precision and coverage. They are the standard measures in the document classification community and this allowed us to make a direct comparison between our results and those in [14,15]. As the goal of our first set of approaches was to construct a system with good precision and then extend its results to obtain full coverage, we decided to examine both measures and not apply their aggregation (like the F measure). Precision is defined as the ratio of the correctly classified (disambiguated) test gene names and the number of total test examples for which the disambiguation method could make a decision, while coverage is defined as the ratio of test instances with a decision and the number of total test examples. Simply put,

$$Prec=\frac{n_{c}}{n_{d}},Cov=\frac{n_{d}}{n_{a}}$$

where *n*_*c *_is the number of correctly disambiguated examples, *n*_*d *_is the number of cases where a decision was made, and *n*_*a *_is the size of the total test set.

### The path between the test and train articles

In our first approach we examined how strong the co-authorship was between the test article and the train articles. The strength of the co-authorship can be measured as the distance between two nodes in the inverse co-author graph. When two nodes are neighbours the two articles have a mutual author. When a node can be reached in two steps, starting from a node means that the two articles have no mutual authors, but some of the authors have a mutual publication (excluding the two articles in question). We looked for the shortest path from the test node to each train example in the inverse co-author graph. Among the closest training points (we gathered all training samples which had the same minimal distance) a majority voting was applied i.e. we made a disambiguation decision in favour of the gene with the closest labelled nodes. Table 1 lists the precision and coverage values we obtained by this method using non-weighted path lengths. A coverage over 90% was achieved on the mouse, fly and yeast datasets by just considering the neighbours of the test nodes, which implies that test nodes and most of the train nodes have a co-author. Significantly fewer articles deal with these organisms than with human and these articles can be processed in a higher coverage by the Entrez group.

**Table 1:** Results obtained using the path-length-based method. Column 1 lists the maximal path distance allowed for each given experiment. The results are presented in a Precision – Coverage format.

Distance Limit	Human	Mouse	Fly	Yeast
1	100%–44.35%	99.88%–97.59%	99.84%–92.19%	100%–99.26%
2	100%–49.19%	98.67%–99.32%	94.58%–97.72%	100%–99.26%
3	85.29%–82.26%	98.64%–99.51%	94.44%–98.10%	100%–99.26%

In our experiments we found that if there was a path between the test node and one of the train nodes (this is true in over 90% of the cases) its length was at most 3. We did not examine this property on the complete graph, but – interpreting training and test nodes as a random sample of node pairs from the graph – we can suppose that the average minimum path length between nodes (articles) is surprisingly small (3 or 4).

### Filtering and weighting of the graph

Table 1 tells us that the noise is considerable in cases where the distance between the closest training node and the test node is 3. We tried to eliminate the noise of these distant training points hence we left out the less reliable edges from the graph. Our hypothesis was that the authors who have a large number of publications do not have a bigger influence and correspondence in articles, hence the edges originating from them are less reliable. To test this hypothesis we ignored the last 10% of the authors from each article, and then repeated each experiment by ignoring those authors who had over 20, 50 or 100 MedLine publications. We investigated two edge-weighting methods on the human dataset along with the filtering process. We calculated the weight *w *for each edge as a function of the number of mutual authors of the two given articles like so:

$$w=\sum \frac{2}{\left|A\cap B\right|}/\min\left(\left|A\right|,\left|B\right|\right),$$

where *A *and *B *are the sets of the authors of the articles. To get an aggregated, weighted distance for a path we summed the edge-weights (*D*_*sum *_= ∑_*i *_*w*_*i*_) or used the minimum of the edge-weights, i.e. the bottleneck of the path (*D*_*min *_= min_*i *_*w*_*i*_).

After calculating the weighted path lengths for each train node we chose (instead of the closest training examples' majority voting) the label of the node with the maximal weight as the final disambiguation prediction.

**Figure 1:**
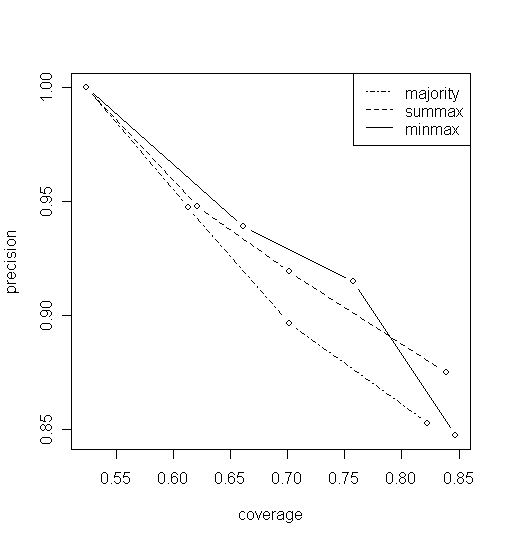
**Precision-coverage curves on the human GSD dataset**. The three curves represents different weighting strategies and their points for different levels of filtering of the inverse co-author graph. The authors who had over 100, 50 or 20 MedLine publications were ignored yielding 3 points on the precision-coverage space, while the fourth point of each curve shows the case without any filtering.

The different degrees of filtering resulted in different precision and coverage value pairs. Figure 1 shows the precision-coverage curves obtained using the three weighting methods (i.e. non-weighted, *D*_*sum *_and *D*_*min*_). According to these results, ignoring more authors from the co-author graph yields a higher precision but at the price of lower coverage. Thus this filtering approach is a parametric trade-off between precision and coverage. A 100% precision can be kept with a coverage of 54.42% while the best coverage achieved by this method was 84.67% with a decrease in precision to 84.76%. The difference between the performance of the three weighting (or non-weighting) methods is significant. The right choice of a method can yield a 2–3% improvement in precision at a given level of coverage. The minmax method seems to outperform the other two, but it does not perform well on the unfiltered graph hence we cannot regard it as the ultimate 'winning' solution here.

### Automatic expansion of the training set

The absence (or small number) of training examples in several cases (especially on the human evaluation set) makes the GSD tasks intractable. To overcome this problem, we extended the labelled set automatically by articles based on the inverse co-author graph. We assumed here that the probability of an author dealing with the same gene in more articles is higher than the probability of dealing with different genes which share an alias. Thus we looked for gene aliases among the articles of the authors and hoped that they used a synonym (or long form) of the target gene name. For example, *CASPASE *in *PMID:12885559 *can refer to genes with EtrezGeneID *37729 *or *31011 *and the document does not contain any synonym belonging to them. One of the authors (*McCall K*.) has two other publications *PMID:999799 *and *PMID:9422696 *which contain *DCP-1 *(EntrezGeneId *37729*), so we assumed that *CASPASE *refers to *DCP-1 *in the test abstract. Our assumption is questionable but as our experiments show it is true in over 90% of the cases.

We labelled each article in the neighbourhood of the test node with a gene identifier if a synonym of the target gene name was found (with exact string matching) in the document. Note that the test abstract (distance 0) can also contain synonyms of the target gene name. In these cases, we made a decision based on this information as well (the special case of distance 0 is equivalent to the disambiguation procedure described in [13]).

After this expansion we made the disambiguation decision via the non-weighted majority voting method on the new set of train samples. Table 2 shows the precision and coverage values we got with this procedure on the four datasets. These values tell us that the articles with a distance of two hardly ever contain gene aliases, which leads to a slight improvement in the coverage rate. We should add that there is a strong statistical connection between the achieved coverage by this method on the particular organism and the size of the available synonym list and labelled train sets.

**Table 2:** Results obtained using the automatic labelled set expanding heuristic. Column 1 refers to the maximal distance allowed in the path finding phase. The results are presented in a Precision – Coverage format.

Distance Limit	Human	Mouse	Fly	Yeast
0	93.3%–12.11%	96.28%–8.57%	100%–7.06%	83.70%–10.23%
1	92.56%–32.82%	91.41%–18.82%	96.56%–10.78%	69.75%–18.79%
2	91.53%–37.88%	91.31%–20.07%	96.56%–10.78%	69.75%–18.79%

We combined the two co-author graph-based methods (minimal path finding and training set expansion) to exploit the advantages of both via the following strategy: when there is at least one training node in the neighbourhood of distance 3 of the test node on the filtered graph, we accept the decision of that model. If there is no such close train node we try to label new documents with the synonym list and make a decision based on these automatically labelled instances. We got some results by applying two filtering and weighting procedure combinations, one yielding a maximal precision and the other a maximal coverage. The precision and coverage values we got of the combined co-author based method can be found in Table 3.

**Table 3:** Results obtained using the combined co-author-based methods

Method	Human	Mouse	Fly	Yeast
With max precision	100%–52.42%	99.76%–97.80%	99.59%–92.42%	100%–99.25%
With max coverage	84.76%–84.67%	99.48%–98.74%	97.94%–95.68%	100%–99.25%

### Achieving the full coverage

In a real world biomedical application the aim is usually to make a disambiguation decision on every gene mention found. As the last rows of Table 2 and 3 make clear, the maximum coverage which can be achieved by our best inverse co-author graph based methods is about 85% on human (and over 98% for the other 3 species). In the last part of our experiments we investigated what effect our co-author graph based heuristics has in a gene disambiguation system which runs on 100% coverage.

We employed two methods, namely the similarity-based procedure introduced by [12] and a supervised machine learning (employing the C4.5 decision tree) approach. We used information provided by MedLine as features including the MeSH headings and information about the release of the articles, the journal title, and the year of publication. Table 4 summarises the results of these two methods applied separately and in combination with the co-author-based heuristics. In this final hybrid system we first applied these two co-author graph-based procedures with filtering to get the highest precision. Then as a second step, we applied a similarity or machine learning technique on the instances where the first step could not make any decision.

**Table 4:** Overview of systems which aimed at full coverage. The most frequent sense was used as the baseline method. We represent the results of Xu et al by using MeSH codes in the second row for the sake of comparability. The results of a C4.5 decision tree using the MeSH features are present in the third row. The systems of the two last rows first apply the combined co-author graph based heuristics and when they cannot decide they use the supervised prediction of the cosine similarity metric or the decision tree.

Method	Human	Mouse	Fly	Yeast
Baseline	59.3%–99.1%	79%	66.7%	65.5%
Xu et al [14, 15] MeSH	86.3%–94.4%	90.7%–99.4%	69.4%–99.7%	78.9%–98.4%
Decision tree	84.68%–100%	90.90%–99.84%	72.53%–99.85%	74.49%–100%
Co-author heuristics + similarity	91.87%–99.19%	98.54%–99.75%	97.20%–100%	94.15%–99.70%
Co-author heuristics + decision tree	94.35%–100%	98.85%–99.91%	96.05%–99.85%	99.63%–100%

The first row of Table 4 lists the precision and coverage values of a baseline method. As a standard in WSD, we used the baseline of choosing the majority sense (the gene having the most training examples) of each gene mention.

¿From a supervised learning point of view the co-author graph-based heuristics eliminate 80% of the errors (decreasing the average error from 18.67% to 4.5% for the similarity measure and from 19.85% to 2.8% for the decision tree), while from the co-author graph point of view the doubtful examples can be predicted with an 80% precision by supervised techniques, thus yielding a full coverage with an aggregated precision of 97.22%.

## Discussion

### Differences among species

There are quite significant differences among the tasks of the given species. The human GSD evaluation set is without doubt the most difficult one for the co-authorship-based approaches because of the extremely large number of articles which focus on this organism and the relative modest number of average training samples available. The co-authorship method achieves precision values over 99% with a coverage of over 92% on the other three datasets. The final results with a complex method (co-authorship-based heuristics along with supervised techniques) correlate with the baseline values (and the ones simple supervised methods) i.e. mouse is the best performing one and a lower precision is obtained on human and fly. The final results on yeast are surprising as baseline methods on this dataset performed the worst but achieved the best results when the co-authorship-based methods were applied (and in the final one as well). We think that this is because of the small amount of articles which focus on this organism, which might imply a smaller author society with stronger relationships.

### Features and methods used

In our experiments we used several kind of features. The main contribution of this work, the path-length in the inverse co-author-based method, just uses the authorship information of the whole MedLine corpus and some manually annotated abstracts (by the Entrez group). The extension of the training set based on the co-author graph and synonym lists is one step closer to the "classical" context-based approaches – namely looking for gene names in the text of the abstracts. This method can be regarded as a generalization of the one in [13] because we search co-authored documents as well, but it is less sophisticated those described in [12] and [14], both of which use external general MeSH term indexing software. In the final supervised learning phase we used a feature set which included MeSH headings (manually annotated in the MedLine), the title of the journal and the year of publication but we did not make use of the text itself. There were several reasons for this. First of all, the manually added MeSH headings represents very well the biological concepts of the article in a normalised and disambiguated way. Second, the empirical results of [15] on two evaluation sets shows that using the words of the text along with MeSH headings could not achieve any significant improvement. We also examined the potentials of the combined usage of headings and text (we lemmatised the text and ignored stop words) in preliminary experiments but no significant improvement was found either hence the text itself was left out for time complexity reasons.

The difference between the baselines and the purely supervised models and the difference between supervised models and final models which employ co-author graph-based heuristics are statistically significant, due to the McNemar's test with a *p *< 0.05 confidence level, but the difference between the two supervised models was below the statistical level of significance. This holds true for the cases of their usage in the final cascade systems as well. The decision tree (when sufficient amount of training data is available) can differentiate the features in a more sophisticated way than the vector space model can. Furthermore, the decision tree can learn complex rules like "the papers released before 2002 and containing Mesh code X but not containing Mesh code Y are...". However, with these complex modeling issues it could not achieve a statistically significant difference compared to the similarity-based approach. This could be because of the small training sets and overfitting. But we suggest using decision trees because its learnt model is human readable so a domain expert can understand and modify it when necessary.

Our results are directly comparable just to Xu et al's results. Table 4 lists the situation where just the features embedded in MedLine were used by both systems, but we achieved better results (with an average precision of 9.5% together with an average improvement of 1.5% in coverage) than the best system of Xu et al [14,15], who employed external automatic annotation tools (MetaMap and BioMedLee) as well.

### Limitations of the approach

The most obvious limitation of our co-authorship based approach is that it is dependent on a training set derived from manual disambiguated annotation by the Entrez group. On viewing Table 1, we see that if the number of annotated articles were higher the GSD task would become a trivial one. There are two factors of the graph construction approach which seem to be negligible but nevertheless deserve a mention here. First, an edge is drawn between nodes because of string matching of the author names. Of course, the names of the authors are also ambiguous as two authors with the same name does not necessary mean they are one and the same person. Second, there should be author-gene pairs which occur in just one publication. In these cases the inverse co-author graph could not help and contextual information has to be taken into account.

When we analysed the misclassified entities we found that most of the errors of two co-author graph-based methods could be eliminated by a sophisticated synonym matching algorithms. Our simple string matching approach, it transpires, has two main shortcomings. It does not handle the spelling variants of the gene aliases (an excellent work handling this task is [4]) and it does not deal with embedded named entities i.e. it matches gene names that are just a substring of a longer name like the name of a protein. The errors of the supervised systems (both the similarity-based and the decision tree-based ones) could probably be eliminated if bigger training sets were available.

## Conclusion

In this paper we examined the utility of co-authorship and experimentally demonstrated the utility of co-authorship analysis for the GSD task. Our hypothesis was that a biologist refers to exactly one gene by a fixed gene alias, and in experiments we found evidence for this. Moreover, we found that a disambiguation decision can be made in 85% of the cases with an extremely high precision rate (99.5%) by just using information obtained from the inverse co-author graph. If we need to build a GSD system with a full coverage we can incorporate the co-authorship information into the system and by doing so eliminate about the half of the errors of the original system.

Based on the promising results obtained so far from our study, we suppose that for abstracts the co-authorship information, the circumstances of the article's release (the journal, the year of publication) and a graph constructed above, can all be crucial building blocks for a sophisticated similarity measure among biological articles and therefore the methods introduced here ought to be useful for other biomedical natural language processing tasks as well. For example, we can reasonably assume that a biologist or biologist author group usually deals with the same special species. Hence a co-author graph-based method could be a powerful tool in the identification of the organism dealing with in an article. In addition, all text classification and clustering tasks can achieve better results with a sophisticated similarity measure. Besides the biological named entity disambiguation tasks (which is also a document classification task), a task could for instance be one for target disease identification or protocol detection.

## Methods

### Evaluation sets used

We evaluated our methods on four different GSD datasets (human, yeast, fly and mouse) which were derived from the two BioCreative Gene Normalisation challenges by [14] and [15]. The evaluation set contained the PMIDs and word forms of the test instances (target words) along with a list of possible gene identifiers (senses) which can be referred to by this name. The most important statistics of these evaluation sets are listed in Table 5.

**Table 5:** The characteristics of the evaluation sets used

Organism	# of test cases	Avg # of senses	Avg size of train set	Avg # of synonyms available
Human	124	2.35	122.09	12.36
Mouse	7844	2.33	263.0	5.36
Fly	1320	2.79	35.69	9.51
Yeast	269	2.08	11	2.32

We downloaded the manually annotated 'gene2pubmed' file from the Entrez website [3] to obtain some labeled articles for each gene. We handled each test instance – along with a given train set – as a separate decision task. The size of the training set (amount of available labelled abstracts) varied from 3 to 500 (there were several gene identifiers with an empty labeled set), which resulted in simpler and harder tasks.

### The construction of the inverse co-author graph

To get the inverse co-author graph we downloaded (in April of 2007) all MedLine abstracts, which contained some 11.7 million instances. We could not construct the whole graph due to space and time restrictions, but we constructed the subgraph of each test example surroundings (nodes reachable in five steps). The number of articles reached in 3 steps (7.2 million for human, 0.7 million for mouse, 0.7 million for yeast and 50 thousand for fly) gives an indication of the amount of studies dealing with each species in question and helps explain the difficulties we had when processing the human dataset.

### Path to the labelled abstracts

To get the distance values we started a breadth-first search from the test node until the closest training nodes (or limit of distance 5) were reached. We kept each node whose distance was minimal and ignored every other training example which had a bigger distance value. The disambiguation decision was made on the majority voting of these closest labeled abstracts, i.e. the gene (sense) that had the most known instances among the closest articles was selected. We found that the training samples obtained in one or two steps were trustworthy, but when the distance of the closest labeled node was three the information we got became noisier.

### Searching for aliases of the target gene name

To get new automatically labeled examples, we made use of the synonym lists provided by the organisers of the BioCreative II task [16] for the human task and the lists of extracted synonyms from the Entrez 'gene_info' file [3] for the mouse, fly and yeast tasks. These lists contain several aliases (synonyms) for each gene. We used the union of these lists of each gene among which the disambiguation is done (we removed those aliases which were ambiguous among the genes in question). These lists are not complete and do not contain every spelling variant but they still proved quite useful in our study.

We gathered all documents which were reachable in at most two steps from the test node in the co-author graph and which contained one of the synonyms of the genes in question. Here we performed exact string matching. Handling the spelling variants or fuzzy matching could further extend the automatically labeled article set. We then labeled those articles with the gene identifier whose alias was found. As the test article sometimes contained synonyms as well, we labeled these cases (with a distance of zero) based on the alias.

### Supervised techniques

There are several cases where the inverse co-author graph based methods cannot make any decision. In a real world biomedical language processing task the goal is to make a clear choice from among several possible meanings in each particular case. In order to achieve a 100% coverage and to examine the behaviour of the graph-based heuristics as a part of a complex GSD system, we applied the following two supervised learning procedures:

• We chose the gene with the maximal cosine similarity between the test article and the centroid of the training samples belonging to a given gene (gene profile). This method was used earlier by [14] and we re-implemented it for the sake of making a comparison between their approach and ours.

• We trained a C4.5 decision tree [18] from the WEKA package [19] on the training examples and accepted its forecast on the test example as a final disambiguation decision. We used the default parameters of the tree learning. Spending some time fine tuning parameters could further improve our results. We employed decision trees (from among the great range of machine learning techniques available) for two reasons. First, this technique is designed to handle discrete features (as in our situation) efficiently and the second was that the learned models are human readable hence human experts can verify or modify them later on if they so wish.

We employed the same feature set in both cases, each feature used being available in the MedLine database. The features we chose to apply were the MeSH headings, the journal title, where the article was released, and its year of publication. We did not use any external knowledge or tagging tool, however.

## Authors' contributions

RF wrote all the software needed to carry out the analysis outlined in this paper and is responsible for the design and interpretation of the study.
